# Indo-Pacific bottlenose dolphin (*Tursiops aduncus*) habitat preference in a heterogeneous, urban, coastal environment

**DOI:** 10.1186/2046-9063-9-3

**Published:** 2013-02-01

**Authors:** Nardi Cribb, Cara Miller, Laurent Seuront

**Affiliations:** 1 School of Biological Sciences, Flinders University, Box 2100, Adelaide, SA, 2001, Australia; 2 Faculty of Science, Technology and the Environment, University of the South Pacific, Laucala campus, Suva, Fiji; 3 The Whale and Dolphin Conservation Society International, Chippenham, UK; 4 South Australian Research and Development Institute, Aquatic Science, West Beach, SA, 5022, Australia; 5 Centre National de la Recherche Scientifique, Laboratoire d’Océanologie et de Géosciences, UMR LOG 8187, Université des Sciences and Technologies de Lille, Station Marine, Wimereux, 62930, France

**Keywords:** Bottlenose dolphin, Conservation, estuaries, Photo-identification, Management, Habitat type

## Abstract

**Background:**

Limited information is available regarding the habitat preference of the Indo-Pacific bottlenose dolphin (*Tursiops aduncus*) in South Australian estuarine environments. The need to overcome this paucity of information is crucial for management and conservation initiatives. This preliminary study investigates the space-time patterns of habitat preference by the Indo-Pacific bottlenose dolphin in the Port Adelaide River-Barker Inlet estuary, a South Australian, urbanised, coastal environment. More specifically, the study aim was to identify a potential preference between bare sand substrate and seagrass beds, the two habitat types present in this environment, through the resighting frequency of recognisable individual dolphins.

**Results:**

Photo-identification surveys covering the 118 km^2^ sanctuary area were conducted over 2 survey periods May to August 2006 and from March 2009 to February 2010. Sighting frequency of recognisable individual Indo-Pacific bottlenose dolphins established a significant preference for the bare sand habitat. More specifically, 72 and 18% of the individuals sighted at least on two occasions were observed in the bare sand and seagrass habitats respectively. This trend was consistently observed at both seasonal and annual scales, suggesting a consistency in the distinct use of these two habitats.

**Conclusions:**

It is anticipated that these results will benefit the further development of management and conservation strategies.

## Background

Cetacean habitats are typically heterogeneous, comprising a mosaic of patches which differ in their biological and physical properties [1]. Understanding the space-time movement patterns and distribution of organisms within their environments can provide insight into the preference of specific areas [1]; information considered essential in the development of management and conservation initiatives [2]. In this context, bottlenose dolphins (*Tursiops* spp.) are no exception. They occur globally in both temperate and tropical waters [3,4], and are common in coastal waters, in particular estuaries, over a wide range of habitat types, such as seagrass beds, sandy substrates and reefs [5-8]. The occurrence of bottlenose dolphins in different habitats illustrates the ecological plasticity and adaptability of this species [2,9-11]. This highlights the need to understand at the individual and population level the key habitat types and locations they preferentially frequent [12]. This is especially critical for populations frequenting coastal environments, which are increasingly impacted by anthropogenic activities, such as tourism, chemical and noise pollution, habitat degradation, commercial and recreational fisheries and aquaculture [13-19], thus making them more susceptible to threats [20,21].

The Indo-Pacific bottlenose dolphin (*Tursiops aduncus*) is a prime example of a coastal dolphin species with many populations throughout the Indo-Pacific region [22], and more specifically Australia, where they are found in a range of coastal environments such as bays, gulfs, lagoons and estuaries that are often highly urbanised [8,16,23,24]. However, little is still known about this species habitat preference in estuarine locations [16].

In South Australian waters, *T. aduncus* is a known resident, especially in the Port Adelaide River – Barker Inlet estuary, where animals have been recorded year-round over the past 18 years [25]. This area supports a small population of approximately 30 resident individuals as well as visiting non-regular transient animals [25,26]. Field observations indicate no other marine mammals, specifically delphinids, living in direct sympatry with this population. Fur seals and sea lions were, however, punctually observed hauled out within the study site. The Port Adelaide River – Barker Inlet estuary is situated in close vicinity to the city of Adelaide, hence it is highly urbanised and subjected to a variety of anthropogenic activities such as industrial and sewage pollution, recreational and commercial vessel traffic, dredging, urban development and habitat degradation [19,27-31]. As a result this area was proclaimed the Adelaide Dolphin Sanctuary (ADS) in 2005 in order to protect both the resident dolphins and their habitat [32].

Baseline habitat information is, however, still scarce and limited to the presence of bottlenose dolphins being independent of environmental features [8]. This potentially limits the development and implementation of effective conservation and management strategies, hence the long term-survival of this population. This also stresses the need to further understand and monitor the preference of habitats within this area at both the seasonal and annual scales, and to identify potential areas of high occurrence of specific individuals. In this context, the objective of this paper was to use photo-identification to assess whether recognisable individuals were consistently sighted in the same benthic habitat type at both seasonal and annual scales.

## Methods

### Study site

**Figure 1:**
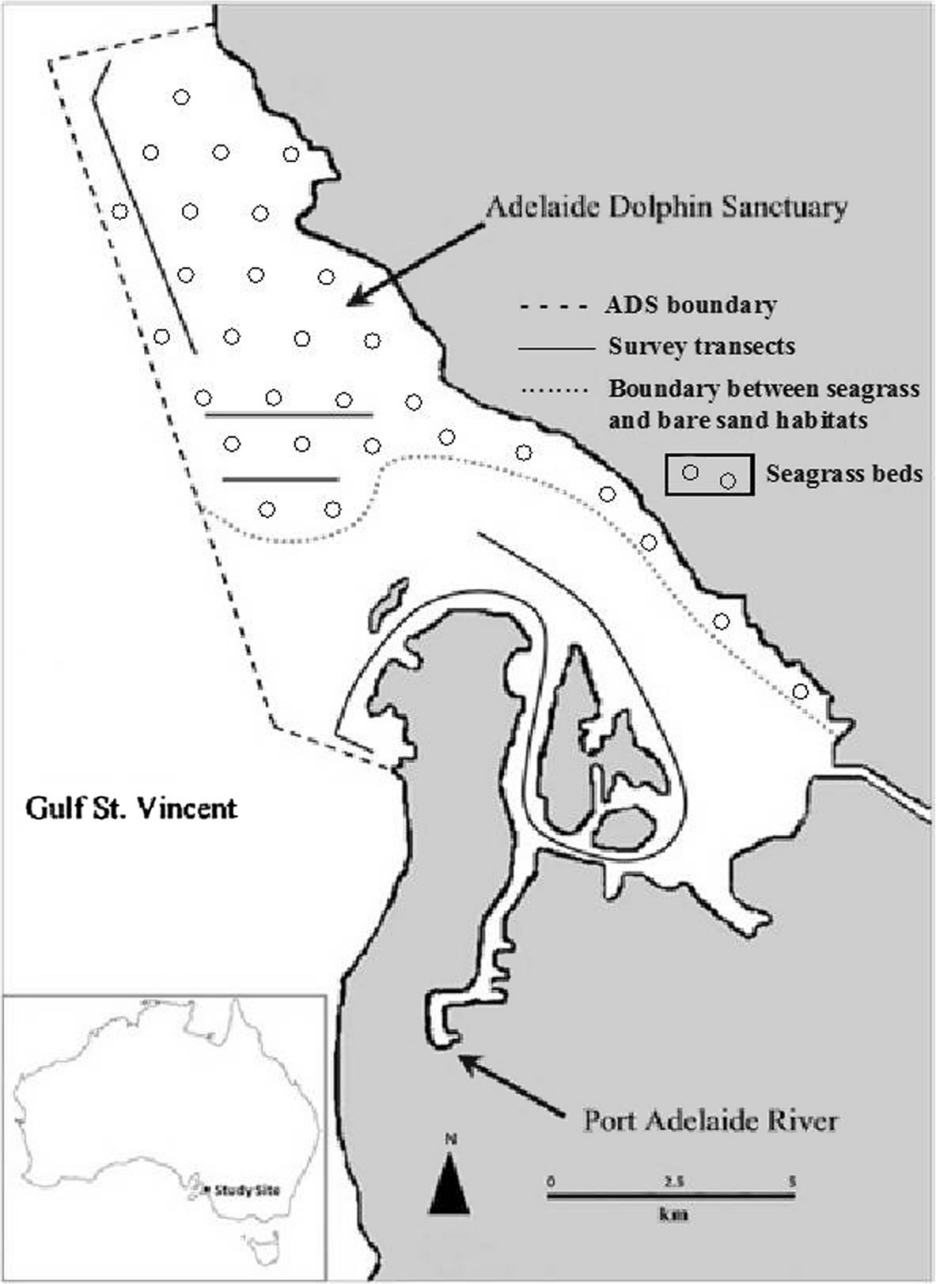
Map of the study area showing the locations of the survey transects (solid black lines), the sanctuary boundaries (dashed line) and the separation between the two benthic habitats (seagrass bed to the North and bare sand to South of the dotted line) in the Adelaide Dolphin Sanctuary, South Australia.

The ADS is situated in the north-eastern region of Gulf St Vincent (GSV), South Australia (Figure 1), located 15 km northwest of Adelaide. This area is characterised by high biodiversity and has both considerable commercial fisheries value and biological significance [33]. The sanctuary area which includes the Port Adelaide River – Barker Inlet estuary and the coastal waters extending northwards out into GSV covers an area of 118 km^2^. In the absence of a map of the benthic habitat in the ADS, we conducted a preliminary sampling survey to assess the nature of the benthic habitat, which showed that the ADS supports two main benthic habitat types that may be used by dolphins (Figure 1). The northern part of the sanctuary extending into the open, unsheltered waters of Gulf St Vincent is characterised by the presence of seagrass beds (predominantly *Posidonia*, *Zostera* and *Heterozostera* sp.; [30,34]. No seasonal fluctuations in seagrass coverage were observed. In contrast, the southern area of the sanctuary consists of shallow sheltered estuarine waters and narrow channels, bordered by mangrove forest, which are essentially devoid of vegetation such as seagrass and attached algae and consist predominantly of bare sand [35]. There is a distinct separation between these two habitat types from the mouth of the estuary out into gulf waters due to the presence of a seasonal sand bars, which constantly change the dynamics of the environment. Water depths in both habitat types range from 0.5 to 6 m, they increase in depth ranging from 10 to 17 m in the dredged shipping channel of the Port Adelaide River. 

### Data collection

Photo-identification data from the ADS were collected between the 5 May and 30 August 2006 and 6 March 2009 to 6 February 2010 (Table 1) following the same methodology. Survey transects were designed to provide both even and representative coverage of the sanctuary and the two benthic environment types found here. Specifically, four transects were used to survey the area (Figure 1). Surveys were always conducted at steady speed of 12 knots aboard either a 6 m rigid-hulled inflatable vessel powered by a 70 HP outboard engine, or a 5 m vessel powered by 70 HP outboard motor and were carried out at a Beaufort Sea state of less than 3, under daylight conditions, between 7:30 am and 3:00 pm and fluctuating tidal conditions. Whilst on transect a constant watch for dolphins was maintained by two observers who scanned the water with the naked eye ahead and to 90° either side of the transects. As boat access was limited in the estuary due the presence of exposed intertidal mud flats not accessible by dolphins and seasonal sand bars, sighting visibility was restricted to 200 m either side of the transect. Upon sighting an individual or group of dolphins (i.e. all animals within a 100 m radius of each other; [12]) the survey effort was ceased to record the time of the sighting and the number of dolphins present. The vessel was then moved as close to the location of the initial sighting as possible to determine the benthic environment type and record the GPS location. Benthic environment type was determined by visual analysis, as the bottom was visible due to the shallow nature and good water clarity. Note that in waters deeper than 10 m the bottom was not visible from the surface. Specifically in the dredged shipping channels of the Port Adelaide River, preliminary benthic sampling consistently showed the benthos to be devoid of vegetation. The benthic environment type was therefore defined within the study area by the presence of seagrass or bare sand. Once the benthic environmental data was recorded the vessel approached the individual or group and it was then endeavoured to photograph as many of the dorsal fins of the animals present as possible [36,37]. A Canon EOS 350D digital SLR with a 75–300 zoom lens was used to take all photographs. Encounters (i.e. an interaction with an individual or dolphin group; [12]) were restricted to a maximum period of 20 minutes in order to attempt to minimise disturbance to the group or until all individuals in the group were photographed. The vessel then returned to the transect and continued until the transect was completed or all of the study area had been surveyed. 

**Table 1:** Number of survey days shown as a function of both season and photo-identification survey periods

**Season**	**Photo-Identification Survey Periods**	
	**Survey Period 2006**	**Survey Period 2009 – 2010**
Spring	1	4
Summer	2	5
Autumn	3	6
Winter	4	8

### Photo-identification analysis

Photo-identification of bottlenose dolphins relies on the matching of distinctive dorsal fin features, such as nicks and notches present on both the trailing and leading edges of the fin, and tip [36,37]. Photographs were assessed for photographic quality (e.g. focus, clarity, contrast, angle, portion of the fin visible and the percentage of picture filled by the fin) and graded according to quality (excellent, average, poor) using Adobe *Photoshop Elements 5.0* imaging software. Only those photographs considered to be of excellent quality were included in the analysis. Poor quality photographs were always discarded from the analysis. Photographs were checked systematically against each other to develop a master catalogue of recognisable individuals and to determine the number of re-sights. The individuals not matched with animals previously recorded were given a unique identification number and added to the catalogue.

### Data analysis

The statistical package PWAS for Windows, version 18, was used for all statistical analysis. As the data failed to meet the assumptions of normality (Kolmgorov-Smirnov test, p < 0.05), non-parametric tests were therefore used to make comparisons between data sets. In order to explore the habitat preference of bottlenose dolphins in the ADS the resighting frequency of individuals (i.e. the sighting frequency of recognisable individuals seen at least on two or more occasions) was estimated for each benthic habitat type. Resighting frequencies were also assessed to identify potential habitat preference between seasons, defined as spring (September - November), summer (December - February), autumn (March - May) and winter (June - August), and years. Additionally, the resighting frequencies were examined to identify habitat preferences on an individual level. Sighting frequencies between habitats were compared using the χ^2^ test [38]. Specifically, our survey equally covered the two habitat types; hence we compared the observed habitat preference frequencies to theoretical frequencies (50% – 50%).

## Results

### Survey and photo-identification effort

**Figure 2:**
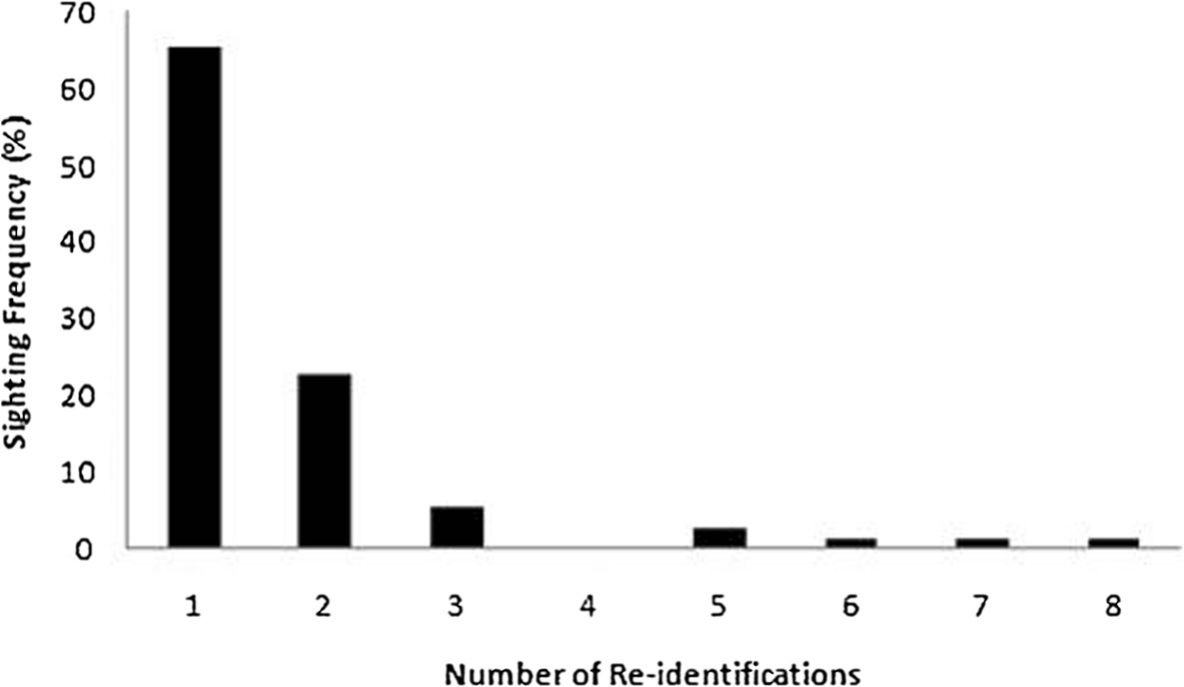
Sighting frequencies for individual dolphins identified in the Adelaide Dolphin Sanctuary in 2006 and between 2009 and 2010.

Twenty two survey days were completed during the two study periods (Table 1). An individual or group of dolphins were sighted on 126 occasions, which resulted in a total of 1602 photographs, and 502 of excellent quality used in the analysis. Although surveys were conducted on different tidal regimes, no effect of tide on the frequency of dolphin occurrence was ever observed. Note, however, that the microtidal regime [39] of the Adelaide Dolphin Sanctuary (and more generally in South Australian gulfs) is unlikely to affect the dynamics of bottlenose dolphins in contrast to megatidal areas such as Aberdeen harbour [40]. A total of 75 distinct individuals were identified based on permanent dorsal fin markings ranging from tip nicks to trailing and leading edge notches. The 75 distinct individuals photographed during the study were sighted between 1 and 8 times. Forty nine of these individuals (65.3%) were sighted on only one occasion. In contrast, 21 (28%) individuals were sighted on two or three occasions and only 5 (6.7%) were sighted on 4 or more occasions (Figure 2). 

### Habitat preference

**Figure 3:**
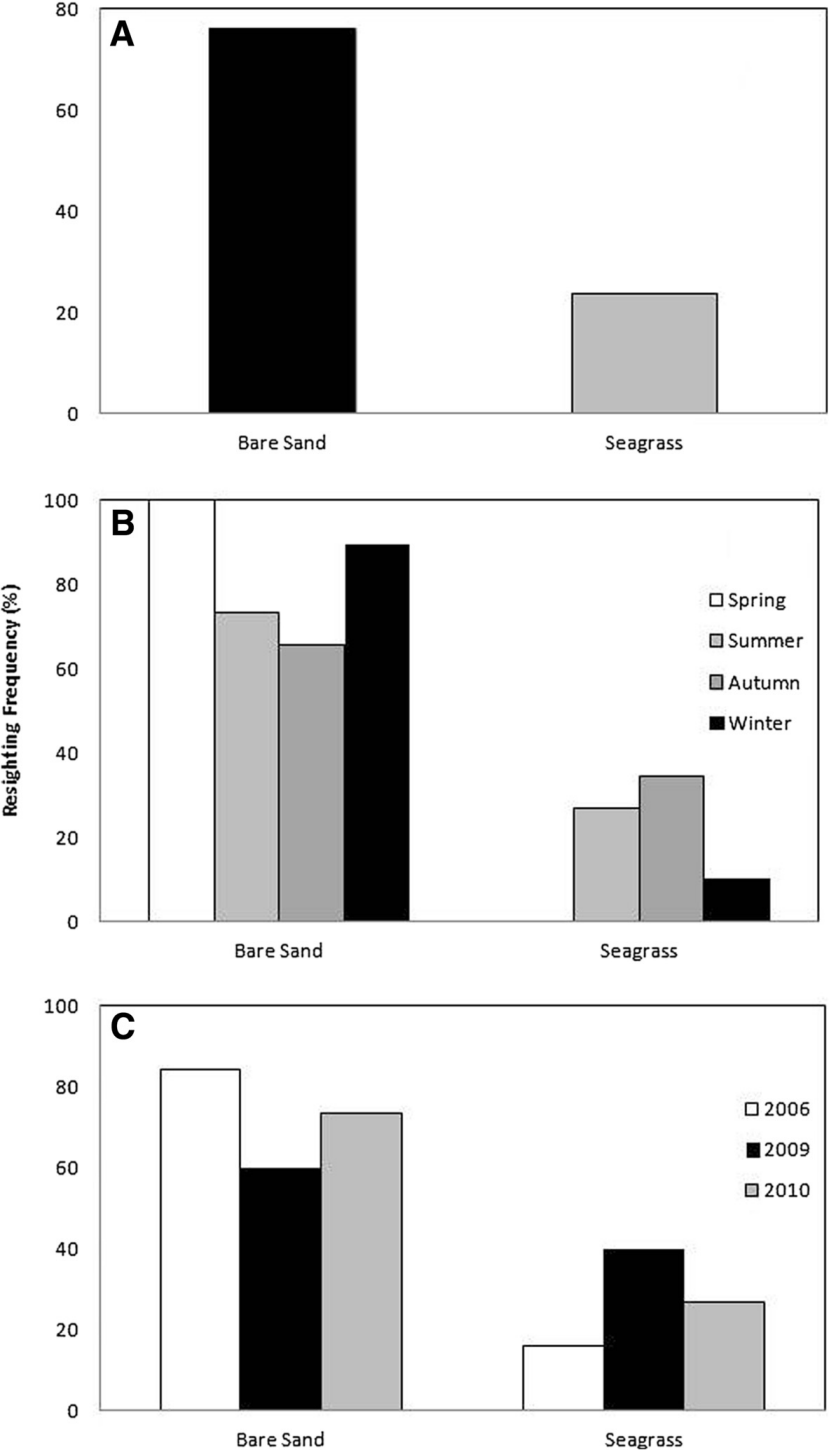
Sighting frequency of recognisable dolphins in the Adelaide Dolphin Sanctuary in relation to habitat type (bare sand and seagrass) over the duration of the whole study (A), and as a function of the season (spring: white; summer; light grey; autumn: dark grey; winter: black; B) and the year (2006: white; 2009: black; 2010: grey; C).

The survey effort equally covered the two habitat types. Bottlenose dolphins were observed throughout the study area over both habitat types. However, the majority of sightings (i.e. 76%, n = 96) was concentrated in the bare sand habitat (χ^2^ test, df = 1, p < 0.05; Figure 3A). A clear seasonal (Figure 3B) and inter-annual (Figure 3C) preference for one of the two habitat types was also observed, with individuals consistently sighted in the bare sand habitat over the four seasons. However, seagrass preference increased from 0 and 10% in winter and spring to 27 and 34% summer and autumn (Figure 3B). The preference for the bare sand habitat was consistent throughout the 3 years of the study (Figure 3C), suggesting that bare sand is the preferred habitat type used by bottlenose dolphins in this area. 

### Individual habitat preference

**Figure 4:**
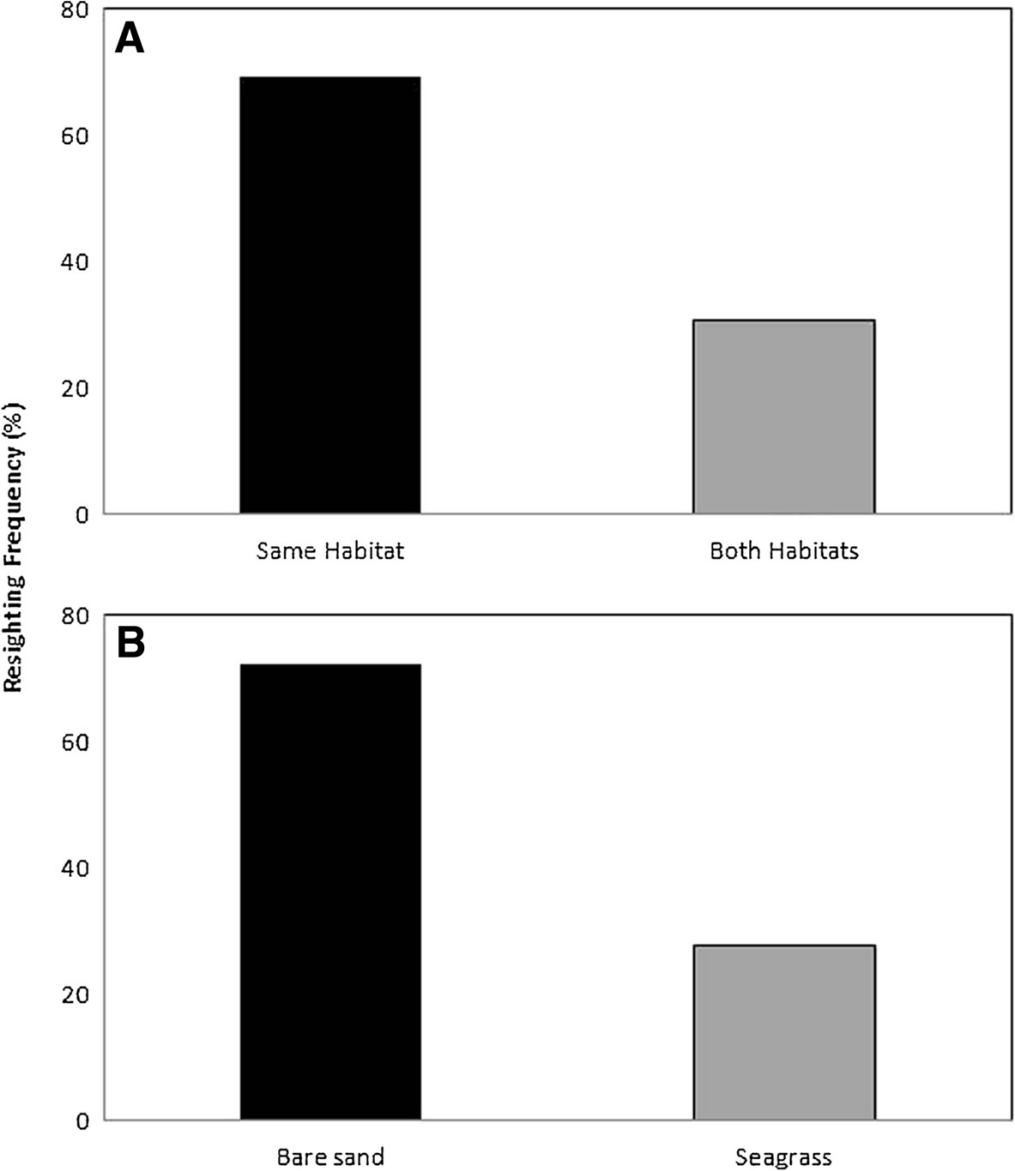
Resighting frequency of (A) individuals consistently sighted in the same habitat or sighted in both habitat types, and (B) only sighted in the same habitat as a function of habitat type.

Recognisable individuals sighted in the ADS on two or more occasions showed a preference for habitat type. Twenty six dolphins were sighted on 2 or more occasions, and 18 of them (69%) were consistently resighted in the same habitat over time. Only 8 individuals (31%) were sighted both over bare sand and seagrass beds (Figure 4A). Additionally, from the 18 animals consistently sighted in the same habitat, 13 (72.2%) and 5 (27.8%) were respectively predominantly (χ^2^ test, df = 1, p < 0.05) resighted in the bare sand and seagrass habitats over time (Figure 4B). 

## Discussion

### Indo-Pacific bottlenose dolphin habitat preference in the ADS

Our observations of dolphin presence and significantly higher sighting frequency in the bare sand habitat (76%; Figure 3A) at both the seasonal and annual scales (Figure 3B,C) and the significantly higher resighting frequency in the same habitat (69%; Figure 4A) are consistent with the previously reported regular occurrence and preference of bottlenose dolphins in one habitat over another [1,6,7,41,42]. While further work is needed to specifically address this issue, our results suggest the presence of a seasonal pattern in habitat preference with an increase in dolphin frequency in the seagrass habitat in autumn and summer (Figure 3B). Seasonal shifts and variations in habitat preference by bottlenose dolphins have also been observed in other locations such as the San Luis Pass (Texas, USA; [43]), the Moray Firth (Scotland; [9]) and the Hauraki Gulf (New Zealand; [44]). However, the occurrence of nearly one-third of the individuals (31%; Figure 4A) over both the bare sand and the seagrass habitats may indicate that a non-negligible proportion of the *T. aduncus* occurring in the ADS have enough behavioural flexibility to use the seagrass beds found in the open waters of Gulf St. Vincent as well as the sheltered waters found in the inner estuarine part of the ADS (Figure 1). More specifically, respectively 72 and 28% of the resighted individuals were observed over the bare sand and the seagrass habitats (Figure 4B). This suggests that the bare sand habitat may be a core area for this population, in contrast to previous work stressing the vital role of seagrass beds for bottlenose dolphins [41,42]. However, further investigation into the behavioural budget of this population is needed to determine how and why these habitats differ in their importance and use.

### Estuaries as important dolphin habitats

**Figure 5:**
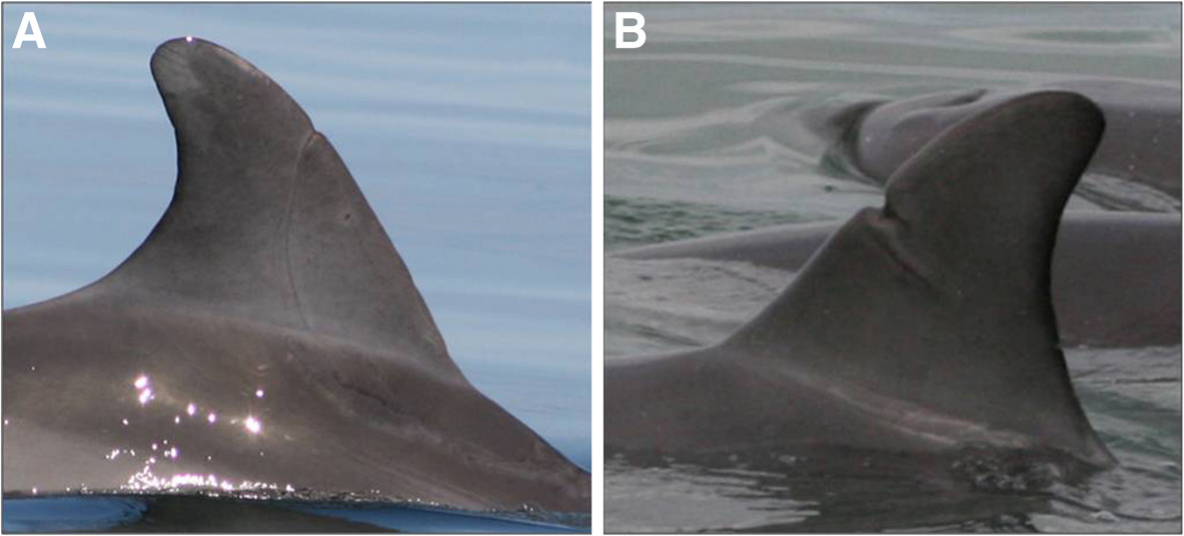
**Examples of both natural (A) and anthropogenic (B) injuries observed on Indo-Pacific bottlenose dolphins photographed in the Adelaide Dolphin Sanctuary. **Natural injuries considered to be inflicted by shark bites are crescent in shape, whilst in contrast those inflicted by anthropogenic causes (e.g. boat strike, entanglements) are usually deeper penetrating ‘slash like’ lacerations.

Our observations of higher dolphin frequency in the bare sand habitat of the Adelaide Dolphin Sanctuary (ADS) further support the importance of estuarine waters for this species [12,16,45-49]. This may be linked to the overall nature of estuaries and their potential for high productivity and prey abundances [50]. Bare sand substrates may also provide a less complex habitat than seagrass in which to feed, particularly as seagrass beds impair their ability to echolocate to find prey [51]. In addition,, the consistent high occurrence of individuals at the seasonal and annual scales in the shallow and sheltered waters of the bare sand habitat (Figure 3B,C) may also be related to threat avoidance, as bottlenose dolphin habitat preference is influenced by shark predation [52]. Specifically, in South Australian waters, dolphins are considered the primary prey of white sharks [53]. Although occasional, the white shark (*Carcharodon carcharias*) and the bronze whaler (*Carcharodon brachyurus*) both frequent the ADS [26]. Despite the relatively low occurrence of sharks in the ADS compared to other locations such as Sarasota (Florida), Moreton Bay (Queensland) and Shark Bay (Western Australia) [26,54-56], one dolphin observed during the study had, a large healed scar on the leading edge of its dorsal fin (Figure 5A). This scar is likely the result of a shark and not other sources such as boat strike, entanglement or other dolphins due to its distinct crescent-shape which contrasts with the deeper penetrating laceration caused by boat strikes and entanglements (Figure 5B), [56]. This suggests that predation may be a potential influencing factor for the high frequency of dolphin sightings in shallow and sheltered waters characterising the bare sand habitat. The bare sand habitat may hence provide a suitable haven from predators, in contrast to the open environment characterising the seagrass habitat. 

### On the influence of sex and social structure on habitat preference

The frequency of the same individuals within the same habitat over time (Figure 4) may be linked to other factors such as social organisation and association patterns [40]. Specifically, bottlenose dolphin habitat preference has been explained by the home range of individuals and the social strategies which individuals or different sexes adopt [1,57]. It has been suggested that protected, shallow and narrow waterways which are geographically further from the open ocean such as the bare sand environment in the present work (see Figure 1), generally promote limited movement patterns and therefore some degree of site fidelity [47,58]. In contrast, individuals found in open habitats have more extensive home ranges and a lesser degree of site fidelity [47,58]. The individuals resighted consistently over time in the bare sand habitat may hence potentially represent resident individuals, and therefore those sighted on fewer occasions in the seagrass habitat may be transients. Additionally, this sighting frequency may be related to foraging or social specific strategies of male and females. Females have smaller home ranges and frequent more areas which provide a higher concentration of resources, such as estuaries that are important for reproduction and calving and the avoidance of predators [47,59]. In contrast, males cover wider ranges than females which has been attributed to female breeding cycles and accessibility [45,59]. As a consequence, the animals sighted consistently in the bare sand habitat might be females utilising local resources, whilst those sighted on fewer occasions in the seagrass may be males searching for females.

## Conclusion

Our results show that bottlenose dolphins in the Adelaide Dolphin Sanctuary occur predominantly in a bare sand habitat. The consistent occurrence and resighting of individuals at both the seasonal and annual scale clearly highlight the importance of the sheltered, bare sand habitat for this population. With a paucity of information available on dolphin habitat due to a lack of monitoring and research in this area, these results provide critical information, which can improve conservation and management strategies previously implemented in the ADS [32]. Specifically, it is recommended to monitor future trends in dolphin spatial and temporal habitat preference, as initiated here through photo-identification surveys. Additionally, due to the presence and potential growth of anthropogenic activities in the vicinity of the ADS, it is critical to understand the details of the seasonal patterns of habitat preference and social activities of bottlenose dolphins that will ultimately help in objectively establishing restricted access to specific core locations of the Adelaide Dolphin Sanctuary. We also stress that the approach presented here may be applicable to other anthropogenically impacted coastal environments, where the identification of dolphin habitat preferences might have critical conservation and management implications. Finally, as the driving mechanisms influencing bottlenose dolphin habitat preferences may differ depending on the intrinsic properties of their environment, such as the nature of anthropogenic activities, coastal geomorphology and bottom topography, further studies are needed to understand habitat choice on both local and global scales.

## Competing interests

The authors declare that they have no competing interests.

## Authors’ contributions

NC and LS designed the research. NC conducted field work and data analysis and wrote the final draft of the paper. CM contributed to previous drafts of the manuscript. LS assisted with statistical analysis and data interpretation and contributed to previous drafts of the manuscript. All authors contributed to the planning and design of the study and have read and approved the final manuscript.
